# Development and Preliminary Evaluation of the OCODES Digital Strategy in Response to Health Misinformation: Nominal Group Technique and Retrospective Usability Evaluation Study

**DOI:** 10.2196/93910

**Published:** 2026-08-14

**Authors:** Emmanuel Echániz-Serrano, Enrique Ramón-Arbués, José Manuel Granada-López, María Teresa Fernández-Rodrigo, Pedro José Satústegui-Dordá, Eva Benito-Ruiz, María Esther Samaniego-Díaz de Corcuera, Natalia Barrio-Forné, Piedad Gómez-Torres

**Affiliations:** 1 Faculty of Health Sciences, Universidad de Zaragoza, Zaragoza, Spain; 2 SALUD_SAPIENF Research Group, Zaragoza, Aragón, Spain; 3 Faculty of Health Sciences, Universidad San Jorge, Campus Universitario, Autovía Mudéjar, km. 299, Highway, A-23, Villanueva de Gállego, 50830, Spain, 34 976 06 01 00; 4 EDU_SAPIENF Research Group, Zaragoza, Aragón, Spain; 5 Servicio Aragonés de Salud, Zaragoza, Aragon, Spain; 6 Faculty of Health Sciences, Universidad de Granada, Ceuta, Spain

**Keywords:** misinformation, health literacy, information dissemination, web-based interventions, nominal group technique, user-centered design

## Abstract

**Background:**

Health misinformation has intensified worldwide, especially in digital environments, where low health and media literacy increase vulnerability. Thus, the MEDIATICH program of the University of Zaragoza launched OCODES (Observatorio Contra la Desinformación en Salud), a participatory digital initiative designed to promote health media literacy and provide evidence-based information through a web platform and social media channels.

**Objective:**

This study describes the co-design and development of the OCODES strategy using 2 sequential nominal group technique (NGT) sessions, and assesses its preliminary usability, preliminary user perception, and user engagement after 6 months of activity as a digital initiative developed in response to health misinformation.

**Methods:**

NGT1 (n=15) aimed to identify and prioritize interventions to strengthen health literacy through an individual yes or no prioritization process, while NGT2 (n=18) translated selected priorities into concrete digital products, including website structure, social media selection, content lines, and interaction mechanisms. In addition, routine user feedback and website analytics data generated through the OCODES digital channels during the first 6 months of activity were retrospectively examined. Usability was explored using the System Usability Scale (SUS), while preliminary user perception was described through 2 study-specific indicators available in the public feedback tools: perceived usefulness (1‐5 Likert scale) and willingness to recommend the website. Google Analytics provided complementary website engagement metrics.

**Results:**

NGT-1 generated 11 interventions grouped into four categories. Digital actions were prioritized as the core strategy: a health dissemination portal and social media interventions in the context of health misinformation each achieved 93.3% (14 of the 15 participants) agreement. NGT-2 defined the digital architecture of OCODES, including a central website and presence on LinkedIn (Meta), X (formerly known as Twitter; X Corp), and Instagram (Meta), and established guiding principles such as transparency, scientific rigor, accessibility, and clear communication. A total of 99 adults provided routine usability feedback (65/99, 65.7% women; mean age 44.7, SD 12.54 y). The mean SUS score was 83.10 (SD 10.09), with 94.9% (94/99) achieving adequate usability (SUS ≥68). Perceived usefulness scored 4.52 (SD 0.71), and 97.9% (97/99) of users who provided feedback indicated that they would recommend the site. Web metrics showed 5769 new users, 1935 returning users, 19,003 page views, a bounce rate of 53.32%, a total of 3.28 pages per active user, and 4.95 events per session, indicating strong initial engagement.

**Conclusions:**

OCODES is a feasible and well-received digital strategy developed through a participatory, consensus-driven process. Its favorable usability indicators, positive preliminary user perception, and encouraging early engagement metrics support its feasibility. However, the current findings do not provide evidence of effectiveness in reducing health misinformation or improving health media literacy. Future efforts should focus on reaching vulnerable populations, expanding evaluation approaches, and assessing long-term impact.

## Introduction

The dissemination of health misinformation represents a substantial and expanding menace to global public health, particularly in the aftermath of the pandemic caused by SARS-CoV-2. The World Health Organization has designated the term “infodemic” to denote the proliferation of information, encompassing both accurate and erroneous content, that impedes the formation of well-informed decisions [1,2].

The dissemination of health misinformation has increased significantly over the past 2 decades, largely due to the democratization of access to information via the internet and the viral spread of unverified content on social media, forums, and websites without scientific validation [3-5]. Digital platforms such as X, Facebook (Meta), YouTube (Google LLC), and Instagram have expedited the propagation of misinformation, eclipsing conventional avenues of scientific and health communication in terms of velocity and scope. The phenomenon has been exacerbated by ease of access, the absence of information mediation or editorial filters, and the tendency of algorithms to prioritize sensationalist content, which displaces evidence-based information [6].

The repercussions of misinformation extend beyond the domain of communication, giving rise to substantial clinical and societal ramifications. This phenomenon has been linked to several adverse outcomes, including but not limited to reduced compliance with treatment regimens [2], discontinuation of preventative measures [7], heightened vaccine hesitancy [8], delays in accessing medical care [9], and elevated rates of preventable morbidity and mortality [10,11]. Moreover, it has been demonstrated that this phenomenon contributes to the erosion of trust in health institutions and health professionals, which in turn hinders the implementation of evidence-based interventions [12].

During the global pandemic, misinformation led to the spread of false narratives about the virus’s origin, the adverse effects of vaccines, and miracle cures without scientific evidence, which undermined trust in science and health institutions [13]. Economic and political interests have used disinformation for commercial gain, particularly in the context of the sale of untested products, or to manipulate public opinion [14]. In this regard, social polarization and the ease with which pseudoscientific ideas can be disseminated have created a digital ecosystem conducive to the spread of fake news and conspiracy theories, with direct consequences for adherence to preventive measures and acceptance of vaccines [15].

It has been demonstrated that exposure to and susceptibility to health-related misinformation is disproportionately affecting people with low levels of education, low incomes, high levels of health-related anxiety, historically vulnerable ethnic populations, and, especially, those with low levels of health media literacy [16-19]. These groups possess an impaired capacity to discern accurate information, encounter greater difficulty when assessing the credibility of sources, and are more inclined to place their trust in social media or nonscientific sources. This, in turn, serves to increase their acceptance of misinformation and medical myths [20,21]. Consequently, inadequate media literacy in health contexts results in a diminished capacity to discern health-related information and the propagation of misinformation [22]. Conversely, feelings of anxiety and stress have been observed to heighten the pursuit of information and render individuals more susceptible to the influence of alarmist or pseudoscientific narratives [19,21]. The digital information environment is characterized by a lack of regulation and adaptation to the needs of these groups, thereby functioning as an independent social determinant that serves to amplify existing disparities [23].

In order to address this challenge, several national [24] and supranational [25] institutions have recommended a multisectoral strategy. This strategy involves the implementation of health literacy campaigns, the establishment of information verification mechanisms, the use of clear and authoritative communication, and the strengthening of trust between the community and health systems [1,12]. The fundamental components of these multisectoral programs should encompass the development of educational resources tailored to different literacy levels, training activities to enhance critical thinking and the ability to analyze health information, in addition to other actions [4]. These strategies are consistent with the primary, secondary, and tertiary prevention approach that has been recommended in the extant literature. This approach ranges from education and the strengthening of individual skills to direct intervention on misinformation in social networks and digital media [26]. In this work, intersectoral collaboration with educational and health institutions, the media, and digital platforms is identified as a key factor in maximizing the reach and sustainability of interventions.

The expected results of these initiatives against misinformation include improvements in the population’s health media literacy, reduced susceptibility to misinformation, increased trust in scientific and health sources, and greater adherence to evidence-based preventive and therapeutic behaviors [27,28]. Some of the activities carried out by the research group affiliated with the University of Zaragoza SAPIENF (B53_23R), and more specifically, its Health Media Literacy Program (MEDIATICH), fall within this context and these objectives. This is a community made up of academics, health professionals, and communication professionals, whose founding objective is to combat misinformation in health, increase health literacy, promote healthy habits, and encourage critical thinking in health. In order to achieve these goals, this alliance proposes initiatives for scientific dissemination and the debunking of misinformation, the creation of educational resources, and training programs for professionals, among others. One of its main initiatives is the OCODES (Observatorio Contra la Desinformación en Salud). This is an open, collaborative, and participatory digital space (website and social networks) where accurate health information is considered a common good. This paper delineates the methodology used in the conceptualization and implementation of the OCODES strategy and reports routine indicators of website usability, preliminary user perception, and initial engagement as indicators of feasibility rather than effectiveness in reducing health misinformation.

## Methods

### Study Design and Data Sources

This paper is a descriptive report of the development and preliminary operational evaluation of the OCODES digital strategy. It is based on 2 retrospective data sources. First, the co-design process was reconstructed from internal records of 2 nominal group technique (NGT) sessions conducted during the routine development of OCODES. These records were used to describe the procedures followed, the proposals generated, and the aggregated prioritization results. Second, routine digital feedback and website analytics generated during the first 6 months of normal platform operation were retrospectively extracted as nonidentifiable data and analyzed and reported in aggregate form. These digital data were used only as preliminary indicators of usability, user perception, and engagement. No data were collected prospectively for a research-specific intervention study.

### Conception of the OCODES Strategy

#### Overview

A structured process was followed for the design and implementation of the OCODES strategy. This initiative was led by health professionals and aimed at promoting health media literacy and addressing health misinformation through digital communication channels. The process progressed from the generation and refinement of ideas to their translation into products (OCODES website and social media presence), using 2 sequential NGT sessions to ensure equitable participation, transparent prioritization, and consensus-based decision-making. OCODES was developed within the broader activities of the MEDIATICH program and the SAPIENF research group. Accordingly, several authors had institutional and operational roles in the development and implementation of the initiative, and this paper should be understood as a transparent descriptive report of that process and its initial operational indicators. The procedural information and results reported for both NGT sessions were reconstructed from the internal meeting records generated during the ordinary development of the OCODES initiative.

**Figure 1.**
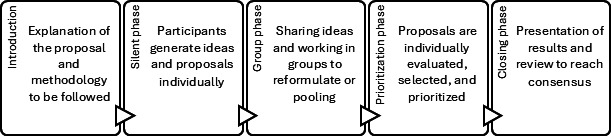
Workflow of a nominal group technique.

NGT is a structured group process that combines group and individual work to facilitate stakeholder participation in generating prioritized and agreed proposals (Figure 1) [29]. The generation of ideas by group members is an individual process. However, these ideas are then worked on collectively in order to decide which ideas should be validated and how they should be prioritized. This process ensures that the influence of dominant participants is minimized and that the voices of all members of the group are heard [29,30]. In order to mitigate the expression of dominance, a series of strategies are used, including the implementation of rotational turns and the articulation of explicit rules of participation. This methodology has previously demonstrated utility in the context of health care process research and design, particularly in scenarios necessitating consensus among diverse professional categories [31,32]. Additionally, its application in defining mass communication and education strategies within the health sector has been highlighted [33].

#### First Nominal Group: Determination of the OCODES strategy

The first NGT (NGT-1) took place in Zaragoza, Spain. It was an in-person meeting of MEDIATICH members, who were selected through intentional sampling based on their experience in health communication, health care practice, and health education. All participants in NGT-1 were members of MEDIATICH and were therefore familiar with the program’s general aims. However, the specific operational configuration of OCODES had not yet been defined at the time of the session. This prior familiarity may have reduced the independence of the first nominal group by orienting discussion toward the broader mission of MEDIATICH. To reduce the influence of preexisting group alignment on idea generation and prioritization, the session incorporated a silent generation phase, a rotating round without debate, structured clarification procedures, and individual binary prioritization. The objective was to prioritize a health literacy strategy, as well as its mission, vision, and values, for OCODES. The group comprised nursing professionals, primarily from universities and health care settings (both primary and specialized care). Fifteen people participated, and their characteristics are summarized in Table 1.

**Table 1.** Demographic data and participant profile (NGT-1^a^; n=15).

Code	Age^b^ (years)	Sex	Preferred pronouns	Field^c^	Focus
A1	45	Female	She	University	Health communication; health literacy
A2	42	Female	She	Primary health care	Community nursing; health education
A3	46	Female	She	Hospital	Clinical nursing; patient safety
A4	38	Male	He	University	Teaching methodology; instructional design
A5	50	Female	She	Primary health care	Health promotion; chronic conditions
A6	44	Female	She	University	Health promotion; clear communication
A7	41	Female	She	Hospital	Quality of care; patient experience
A8	47	Female	She	University	Digital health literacy
A9	35	Female	She	Primary health care	Prevention and community health
A10	52	Male	He	University	Program evaluation; indicators
A11	44	Female	She	Primary health care	Health counseling; easy reading
A12	40	Female	She	Hospital	Therapeutic education
A13	48	Female	She	University	Scientific dissemination; social media
A14	33	Female	She	Primary health care	Young people and health; brief messages
A15	57	Male	He	University	Ethics and editorial policy

a NGT: nominal group technique.

b Mean age (SD): 44.1 (6.35); range 33‐57 years.

c Field of activity reported by the participant.

The session was moderated by a researcher with specific experience in NGT, whose role was limited to process facilitation, wording clarification, time management, and enforcement of turn-taking rules. The moderator explained the rules, the focus question, and the decision-making framework before beginning the ideation process; ensured adherence to rotating turns to avoid dominance and encourage equal participation; and controlled time in accordance with methodological recommendations for consensus studies. To protect neutrality, the moderator avoided evaluative feedback on proposals and ensured that any wording changes introduced during the clarification phase were reviewed with the group before the prioritization stage [29,32].

After sharing the objectives and rules, the moderator presented, as a methodological stimulus, a list of brief, prioritizable interventions supported by evidence to encourage reflection without limiting free ideation. Next, participants were asked to produce a list of proposals individually to improve health literacy among the general population. These proposals were then shared in a rotating round, without debate, to ensure they were recorded and considered consistently. The categories and proposed interventions are listed in Table 2.

**Table 2.** Possible interventions to improve health literacy in the population (reference for ideation and prioritization in NGT-1^a^).

Categories and proposed interventions	Objective:
Digital strategy	
Health information website	To create a reliable, accessible, and user-friendly digital space for the public.
Work to combat misinformation on social media	To provide reliable information and counteract misinformation about health issues.
Creation of digital resources for consultation	To provide practical materials that are always accessible to the public and professionals.
Mailbox or helpline for digital health queries	To provide an accessible channel for the public to ask questions about dubious information and receive evidence-based guidance.
Working with institutions	
Working with institutions or advocacy for health	To collaborate with local councils, regional authorities, health departments, educational centers, companies, and local organizations.
Community work	
Educational work in the community	To carry out direct, face-to-face interventions with the general population, vulnerable groups, educational centers, workplaces, associations, etc.
In-person conferences and workshops in the community	To promote access to reliable health information through in-person educational activities aimed at different community groups.
Free courses for the population	To strengthen health and digital literacy by offering accessible training to improve understanding and critical use of health information.
Training and networking	
School of health activists	To train key individuals, professionals, and organized communities to increase the dissemination of reliable information.
Creation of a network of “health ambassadors”	To develop a community network of trained agents to disseminate accurate information and detect hoaxes in their environments.
Creation of a network of local professional verifiers	To establish a multidisciplinary group responsible for monitoring, analyzing, and verifying dubious content, providing evidence-based responses.

a NGT: nominal group technique.

This was followed by a clarification and grouping phase, in which overlapping ideas were reviewed collectively and merged only when participants agreed that they referred to the same operational proposal. When differences in scope or meaning remained, proposals were retained as separate items. The final wording of grouped items was reviewed with the participants before the prioritization stage.

When disagreements arose during the clarification phase, these were addressed through brief structured discussion focused on wording and conceptual boundaries. If agreement was not reached, proposals were not forced into a single category but were retained as separate items for individual prioritization.

The exercise concluded with an individual binary prioritization process. Each participant indicated, for each proposal, whether it should be considered a priority for the OCODES strategy (yes or no), taking into account previously agreed criteria such as the vulnerability of the target population, potential public health impact, operational feasibility, sustainability, equity, and available evidence. The number of “yes” endorsements received by each proposal was then counted and expressed as the proportion of participants supporting that initiative.

The effective time was distributed as follows: 15 minutes for silent generation; 30 minutes for the round; 35 minutes for clarification and grouping; and 15 minutes for prioritization. Additionally, 10 minutes at the beginning were reserved for formulating the session’s objectives and rules, and a further 10 minutes at the end were reserved for closing and planning the next steps. Thus, the total duration of the session was 125 minutes.

#### Second Nominal Group: Transfer to Products

The second NGT session (NGT-2) was held in Zaragoza, in person. The operational objective was to determine the sections of the OCODES website, select the social networks to be used, and define the content to be published. This included prioritizing topics, establishing social relevance criteria, and deciding on formats, such as short messages and evidence-based papers, as well as interactive resources, such as questions for visitors. Eighteen people were recruited through purposive sampling to ensure the presence of key profiles for design and implementation: 8 health professionals, 5 communication and digital management professionals, and 5 potential users (Table 3). Participants in NGT-2 were recruited specifically for this phase and were not members of MEDIATICH. This sampling strategy was intended to incorporate external perspectives from health professionals, communication specialists, and potential users to inform the design and implementation of OCODES.

**Table 3.** Demographic data and participant profile (NGT-2^a^; n=18).

Code	Age^b^ (years)	Sex	Preferred pronouns	Field^c^	Focus
B1	27	Female	She	Potential user (general population)	Consumption of information on social media
B2	32	Male	He	Digital communication (industry)	Social media; marketing
B3	34	Female	She	Health (primary health care)	Community nursing
B4	31	Male	He	Health (hospital)	Internal medicine
B5	33	Female	She	Health (university)	Public health; misinformation
B6	35	Female	She	Digital communication (agency)	Digital content strategy
B7	37	Female	She	Health (primary health care)	Health promotion
B8	39	Male	He	Digital communication (analytics)	Web analytics
B9	41	Female	She	Health (hospital)	Clinical nursing
B10	42	Female	She	Potential user (general population)	Perceived health literacy
B11	44	Female	She	Health (university)	Teaching; editorial policy
B12	45	Male	He	Health (primary health care)	Community pharmacy
B13	47	Female	She	Potential user (general population)	Web interaction
B14	49	Female	She	Digital communication (industry)	Usability; accessibility
B15	51	Male	He	Health (hospital)	Surgery; patient safety
B16	53	Female	She	Health (university)	Program evaluation
B17	55	Male	He	Potential user (general population)	Interest in evidence and sources
B18	61	Female	She	Potential user (general population)	Easy reading; numeracy

a NGT: nominal group technique.

b Mean 42.0 (SD 8.58); range: 27‐61 years.

c Field of activity reported by the participant.

The session was led by a moderator who was experienced in NGT and digital communication in health, and whose role was limited to process facilitation, clarification of wording, time management, and enforcement of turn-taking rules. To protect neutrality, the moderator avoided evaluative feedback on proposals, applied the same participation rules to all attendees, and ensured that any reformulations introduced during the clarification phase were reviewed with participants before the prioritization stage. Finally, the moderator ensured that all proposals were recorded in full and that individual binary prioritization was conducted consistently across participants.

The session opened with a 15-minute benchmarking block to align best practices and opportunities for improvement based on similar strategies in observatories and institutional channels. This methodological stimulus served to activate critical reflection without constraining ideation, in line with the NGT. This was followed by the NGT sequence itself, with times adjusted to the methodological recommendations. Namely: silent generation of proposals in which each participant wrote down ideas on website architecture, social media, and publication formats with interactive elements (15 min), rotating round without debate (30 min), clarification or grouping to refine overlaps and agree on categories (40 min), and individual binary prioritization (15 min).

During the clarification phase, proposals were merged only when participants considered them conceptually equivalent; when disagreement remained regarding scope or meaning, proposals were retained as separate items. In the prioritization phase, each participant indicated whether each proposal should be considered a priority (yes or no), and the number of endorsements was used to establish the level of support for each option.

In addition, the first 10 minutes were reserved for setting the objectives and rules for the session, and the last 10 minutes for closing remarks and farewells to the participants. In total, NGT-2 lasted 120 minutes.

#### Consensus Criteria and Analysis

In both NGTs, prioritization was carried out individually using a binary endorsement procedure. Specifically, each participant indicated for each proposal whether it should be considered a priority (yes or no). The total number of “yes” endorsements received by each proposal was then counted and expressed as a proportion of the total number of participants. Following standard practice in NGT, consensus was defined a priori as support from at least 80% of participants (ie, ≥12/15 participants in NGT-1 and ≥15/18 participants in NGT-2). This definition was established in advance and explained to participants before the prioritization stage. No weighted scoring or rank-order formula was applied; agreement values therefore correspond directly to the number and proportion of participants endorsing each proposal as a priority. The reporting of the NGT component was further aligned with key principles of the ACCORD (Accurate Consensus Reporting Document) framework for consensus methods, and a checklist mapping the relevant reporting items to this paper’s sections is provided in Checklist 1.

### Routine Digital Feedback and Website Metrics

During the first 6 months of OCODES activity, routine user feedback and website analytics data generated through the OCODES digital channels were available as part of normal platform operation. For this paper, these preexisting data were retrospectively extracted as nonidentifiable records and analyzed and reported in aggregate form as preliminary indicators of usability, user perception, and engagement.

Among the routinely available feedback items, usability was explored through the System Usability Scale (SUS), a tool developed by Brooke [34] and validated in its Spanish version by Sevilla-Gonzalez et al [35]. In odd-numbered questions, 1 point was subtracted from the score corresponding to the user’s response. In even-numbered questions, 5 points were subtracted from the value of the response. Subsequently, all values for the 10 questions were added together and multiplied by 2.5. The final score ranges from 0 to 100, with higher values representing better usability. Based on the literature, this study used a cutoff point of 68 to define adequate usability [34].

In addition to the SUS, two brief study-specific feedback items routinely available through the OCODES digital tools were examined: (1) “Rate the usefulness of our website” on a scale from 1 (minimum) to 5 (maximum), and (2) “Would you recommend the OCODES website to others?” (yes or no). These items were used as preliminary indicators of perceived usefulness and overall user endorsement, rather than as a comprehensive or validated assessment of acceptability.

Website analytics were also obtained through Google Analytics as complementary indicators of initial engagement with the platform. Specifically, the indicators were bounce rate, pages per session, percentage of interactions, average session duration, new and returning users, number of views per active user, and events per session. The bounce rate indicates the percentage of sessions in which the user leaves the site without performing a significant interaction. The pages viewed per active user reflect the average number of pages viewed during a session and allow estimation of the degree of content exploration. The percentage of interactions indicates the proportion of sessions in which the user stays for more than 10 seconds, views more than one page, or activates a conversion event, constituting a relevant indicator of reading quality. Average session duration estimates the time visitors spend on content. New user and returning user metrics allow site reach (ability to attract new visitors) and loyalty (retaining users) to be differentiated. Views and active users were used together to calculate views per active user, a key indicator of internal engagement. Finally, events per session measure the average number of meaningful actions performed by each user (eg, scrolling, clicks, video playback, or downloads), providing an overview of engagement with the content.

These routine feedback and analytics data do not constitute a validated or comprehensive acceptability assessment, and no qualitative feedback, formal accessibility testing, or task-based usability testing was conducted in this preliminary evaluation. As these indicators were derived from preexisting routine digital feedback tools and website analytics, rather than from a research-specific internet survey administered by the investigators, the digital component was not reported as a CHERRIES (Checklist for Reporting Results of Internet E-Surveys)–based web survey study.

### Data Analysis

In the usability analysis of the OCODES website, quantitative variables are presented using the mean and SD, and categorical variables are presented using numbers and percentages. The association between SUS scores and the variables of age, gender, and education level was tested using Spearman correlation, the Mann-Whitney *U*, and the Kruskal-Wallis H, respectively. Statistical significance was defined as *P*<.05. All data were analyzed with SPSS (version 29.0; IBM Corp).

### Ethical Considerations

No formal ethics committee review or waiver was sought for this paper because the work did not involve a prospective research protocol designed to collect data from human participants. Instead, this paper reports a retrospective descriptive analysis of information that already existed before the decision to prepare this academic paper. This information came from two sources: (1) internal records generated during the ordinary participatory development of the OCODES strategy and (2) routine digital feedback and website analytics generated during normal platform operation.

Under Spanish Law 14/2007 on Biomedical Research [36], Article 1.1 defines the scope of the law as including, among other activities, “health-related research involving invasive procedures” (“las investigaciones relacionadas con la salud humana que impliquen procedimientos invasivos”), the processing of biological samples, the storage and movement of biological samples, and biobanks. Article 3(t) defines an invasive procedure as “any intervention performed for research purposes that involves a physical or psychological risk for the affected subject” (“toda intervención realizada con fines de investigación que implique un riesgo físico o psíquico para el sujeto afectado”). Article 12.2(e) states that Research Ethics Committees issue prior reports on “biomedical research involving interventions in human beings or the use of biological samples of human origin” (“toda investigación biomédica que implique intervenciones en seres humanos o utilización de muestras biológicas de origen humano”). The present manuscript did not involve a prospective biomedical research intervention, experimental assignment, clinical procedure, biological samples, genetic data, biobanks, or identifiable health data. For this reason, the activities reported were classified by OCODES/MEDIATICH as institutional development and retrospective routine digital service evaluation, rather than as biomedical research requiring Research Ethics Committee review under Law 14/2007.

The NGT sessions were conducted as part of the ordinary strategy-development and educational activities of the MEDIATICH program, not as research interventions. No data were collected at the time of the sessions for a prospective research study. For this paper, information on the NGT process and aggregated results was retrospectively reconstructed from internal meeting records generated during the routine development of OCODES. After the decision was made to report the OCODES development process in academic form, all NGT participants were informed that the procedures followed and the aggregated results of the sessions would be included in a paper. They explicitly authorized the use of this information for publication, including the aggregated prioritization results and coded, nondirectly identifiable demographic and professional descriptors used to characterize the nominal group panels. No individual statements, quotations, names, contact details, audio recordings, or video recordings were included in the reported materials.

The digital component was also retrospective. It used only preexisting routine feedback and website analytics generated during normal platform operation. These data were not originally collected for research purposes and were analyzed as nonidentifiable records, with the results reported only in aggregate form. The feedback items, including usability and perceived usefulness indicators, were voluntary and available through public feedback mechanisms embedded in the OCODES website. For the purposes of this paper, the authors did not extract, access, or analyze IP addresses, direct identifiers, or traceable personal metadata.

The OCODES, within the MEDIATICH program, acts as the data controller for the platform and is responsible for data governance. Website users were informed through the platform’s publicly available privacy policy and cookie banner that usage data and navigation-derived information could be collected and analyzed to understand audience characteristics, traffic patterns, and overall platform use. The legal basis for processing voluntarily provided personal data and analytics data was user consent, obtained through acceptance of the privacy policy and cookie consent mechanisms. Google Analytics was used in accordance with the platform’s cookie policy.

Personal data, when voluntarily provided by users through contact, subscription, comment, or other website forms, were processed according to the stated purposes of communication, service management, moderation, and maintenance of the relationship established through such forms. These data were stored securely and retained only for the legally established period or until the user requested erasure. Users were informed of their rights of access, rectification, erasure, restriction, objection, and withdrawal of consent through the privacy policy and dedicated contact mechanisms. Users could revoke consent or object to data processing by contacting OCODES through the email address provided in the privacy policy.

Consistent with the General Data Protection Regulation principles on anonymization, the analyses reported in this paper used only nonidentifiable digital data, and results are presented in aggregate form. The reported digital dataset did not allow the authors to identify individual users. Accordingly, the absence of formal ethics committee review was based on the secondary, retrospective, noninterventional nature of the work; the fact that no data were originally collected for research purposes; and the use of preexisting institutional records and routine digital data reported only in aggregated or nonidentifiable form.

## Results

### The OCODES Strategy

NGT-1 generated, refined, and prioritized a set of interventions aimed at strengthening health literacy and addressing health misinformation. Following individual ideation, pooling, clarification, and grouping phases, a final repertoire of 11 interventions was configured, organized into 4 operational categories: digital strategy, working with institutions, community work, and training and networking.

During the prioritization phase, each participant individually indicated whether each intervention should be considered a priority, based on the agreed prioritization criteria. The total number of endorsements for each proposal resulted in the group classification presented in Table 4.

**Table 4.** Results of the NGT-1^a^.

Category and proposed interventions	Agreement with the initiative
Digital strategy, n (%)	
Health information website	14/15 (93.3)
Work to combat misinformation on social media	14/15 (93.3)
Creation of digital resources for consultation	12/15 (80.0)
Mailbox or helpline for digital health queries	11/15 (73.3)
Working with institutions, n (%)	
Working with institutions or advocacy for health	7/15 (46.5)
Community work, n (%)	
Educational work in the community	10/15 (66.6)
In-person conferences and workshops in the community	11/15 (73.3)
Free courses for the population	6/15 (40.0)
Training and networking, n (%)	
School of health activists	9/15 (60.0)
Creation of a network of “health ambassadors”	8/15 (53.3)
Creation of a network of local professional verifiers	7/15 (46.6)

a NGT: nominal group technique.

Overall, the group prioritized digital interventions as the main strategic focus. The health information portal or website and the work to combat misinformation on social media both achieved 93.3% (14/15) agreement. The creation of digital resources for consultation (12/15, 80%) and the digital health inquiry box or helpline (11/15, 73.3%) also received high scores. In the community work category, face-to-face conferences and workshops obtained 73.3% (11/15) in agreement, while general community interventions reached 66.6% (10/15) in agreement. In contrast, free courses for the population received less support (6/15, 40%).

Proposals related to training and networking showed moderate levels of consensus: the school of health activists obtained 60% (9/15) in agreement, the health ambassadors network obtained 53.3% (8/15) in agreement, and the network of local verification professionals obtained 46.6% (7/15) in agreement. Finally, the intervention related to working with institutions/advocacy for health achieved 46.5% (7/15) agreement, similar to some components of professional training.

Based on the interventions prioritized in NGT-1, NGT-2 was carried out. The purpose of NGT 2 was to specify the essential components of the future OCODES strategy, including the selection of digital platforms, publication criteria, and the guiding principles of this project. Participants generated proposals regarding the most appropriate social networks, types of content, priority themes, and mechanisms for interaction with citizens. After grouping and prioritizing these proposals, a strategy was defined around a central web portal [37] and profiles on LinkedIn, X, and Instagram, chosen for their reach, versatility, and suitability for the rapid dissemination of evidence-based health information and timely responses to misinformation.

The group established a set of guiding principles, which were subsequently incorporated into the institutional website, including transparency, absence of personal interests, rigor and scientific evidence, accessibility, usability, and social responsibility in health communication.

Priority topics were also defined based on the needs identified in NGT 1, with an emphasis on public health, self-care, digital literacy, health misinformation, and content related to current social issues. It was agreed to use a dual content model, combining short messages for rapid dissemination and expanded materials backed by evidence. Interactive resources such as open-ended questions, links to verified sources, and citizen consultation mechanisms were also incorporated.

### Routine Usability Indicators and Preliminary User Perception of the OCODES Website

During the first 6 months of operation of the OCODES website and social media channels, routine feedback data generated through the platform’s digital tools were recorded and later analyzed as nonidentifiable records, with results reported in aggregate form. A total of 99 adults provided routine digital feedback, comprising 65 (65.7%) women and 34 (34.3%) men, with an average age of 44.7 (SD 12.54; range 22‐80) years and a predominance of participants with university education (76/99, 76.8%), followed by those with secondary education (20/99, 20.2%) and primary education (3/99, 3%). The usability of the OCODES website was measured using the SUS, obtaining an average score of 83.10 (SD 10.09) points, with a median of 82.50 (IQR 32.50) points and values ranging from a minimum of 62.50 to a maximum of 100 (Table 5). A total of 94.9% (94/99) of participants exceeded the threshold for adequate usability on the scale (SUS ≥68).

**Table 5.** Usability evaluation of the OCODES^a^ website using the SUS^b^ (n=99).

	Strongly disagree, n (%)	Disagree, n (%)	Neutral, n (%)	Agree, n (%)	Strongly agree, n (%)	Mean (SD)	Median (IQR)	Minimum	Maximum
I think that I would like to use this system frequently	0/99 (0.0)	1/99 (1.0)	14/99 (14.1)	42/99 (42.4)	42/99 (42.4)	4.26 (0.73)	4.00 (2.00)	2.00	5.00
I found the system unnecessarily complex	42/99 (42.4)	52/99 (52.5)	2/99 (2.0)	3/99 (3.0)	0/99 (0.0)	1.65 (0.67)	2.00 (1.00)	1.00	4.00
I thought the system was easy to use	0/99 (0.0)	1/99 (1.0)	6/99 (6.1)	44/99 (44.4)	48/99 (48.5)	4.40 (0.65)	4.00 (1.00)	2.00	5.00
I think that I would need the support of a technical person to be able to use this system	57/99 (57.6)	36/99 (36.4)	4/99 (4.0)	2/99 (2.0)	0/99 (0.0)	1.50 (0.67)	1.00 (1.00)	1.00	4.00
I found the various functions in this system to be well-integrated	0/99 (0.0)	4/99 (4.0)	9/99 (9.1)	50/99 (50.5)	36/99 (36.4)	4.19 (0.76)	4.00 (2.00)	2.00	5.00
I thought there was too much inconsistency in this system	41/99 (41.4)	47/99 (47.5)	9/99 (9.1)	2/99 (2.0)	0/99 (0.0)	1.71 (0.71)	2.00 (1.00)	1.00	4.00
I would imagine that most people would learn to use this system very quickly	0/99 (0.0)	1/99 (1.0)	11/99 (11.1)	48/99 (48.5)	39/99 (39.4)	4.26 (0.69)	4.00 (2.00)	2.00	5.00
I found the system very cumbersome to use	52/99 (52.5)	42/99 (42.4)	3/99 (3.0)	2/99 (2.0)	0/99 (0.0)	1.54 (0.65)	1.00 (1.00)	1.00	4.00
I felt very confident using the system	1/99 (1.0)	1/99 (1.0)	21/99 (21.2)	37/99 (37.4)	39/99 (39.4)	4.13 (0.85)	4.00 (2.00)	1.00	5.00
I needed to learn a lot of things before I could get going with this system	53/99 (53.5)	37/99 (37.4)	7/99 (7.1)	1/99 (1.0)	1/99 (1.0)	1.58 (0.75)	1.00 (1.00)	1.00	5.00
Total	N/A^c^	N/A	N/A	N/A	N/A	83.10 (10.09)	82.50 (32.50)	62.50	100

a OCODES: Observatorio Contra la Desinformación en Salud.

b SUS: System Usability Scale.

c N/A: not applicable.

Subgroup analysis did not reveal significant differences in SUS scores according to gender (U=1037.0; *P*=.615), age (rho=0.012; *P*=.90), or educational level (H=2.687; *P*=.26), with similar usability means in the different educational strata, suggesting a homogeneous user experience regardless of these sociodemographic characteristics. As routine indicators of preliminary user perception, the perceived usefulness of the website obtained an average score of 4.52 (SD 0.71) on a scale of 1 to 5, and 95.9% (95/99) of respondents rated the platform with 4 or 5 points. Consistent with this pattern, 97.9% (97/99) said they would recommend the OCODES website to others. These findings suggest favorable initial user perception, although they should not be interpreted as a comprehensive or validated assessment of acceptability.

### Website Metrics

The overall bounce rate was 53.32%, indicating that just over half of the sessions ended without interaction. The average number of page views per active user was 3.28. The interaction rate reached 46.68%, and the average session duration was 3 minutes and 9 seconds. In terms of audience, 5769 new users and 1935 returning users were recorded, reflecting a high initial reach and a lower, but still significant, level of return. During the period analyzed, there were 19,003 views and 5801 active users, which translates into 3.28 views per active user.

The average number of events per session was 4.95, indicating a high level of interaction through actions such as scrolling, clicking on links, playing videos, or downloading files.

## Discussion

### Principal Findings

This study describes the conception, development, and initial evaluation of OCODES, a digital platform developed to promote health media literacy in the context of health misinformation. The results obtained allow us to discuss, on the one hand, the suitability of the methodology used to design the strategy and, on the other, the initial performance of the initiative in terms of routine usability indicators and preliminary user perception, as well as its potential scope and the future challenges associated with its consolidation and scalability. It is important to note that this study focuses on the development and initial evaluation of the platform and does not assess its effectiveness in modifying misinformation-related outcomes.

### Suitability of the NGT for Designing the OCODES Strategy

The use of the NGT proved particularly suitable for achieving the objectives set out in this study. The NGT allowed for the structuring of a participatory, transparent, and consensus-oriented process [38], minimizing the influence of dominant participants and favoring the equal expression of all voices, as recommended by the methodological guidelines for health consensus studies [39]. This approach was particularly relevant in a context such as health media literacy, where clinical, communicative, educational, and social dimensions converge. The prioritization obtained in NGT-1, which placed digital interventions at the center of attention over other community or institutional strategies, is consistent with the literature that identifies the digital environment as one of the main vectors for the spread of misinformation, but also as a key setting in which evidence-based communication strategies can be deployed [40,41].

A distinguishing feature of the design was the incorporation, in the second NGT, of diverse profiles that included health professionals, digital communication experts, and potential users. This diversity allowed for the integration of complementary perspectives on scientific rigor, language accessibility, digital formats, user experience, and actual information consumption patterns, aligning with evidence that highlights the importance of codeveloping digital health interventions with end users and key stakeholders [42]. In this sense, participatory and user-centered design approaches allow for the identification of barriers, specific needs, and contextual requirements, facilitating the integration of tailored solutions and increasing the likelihood of adoption and scalability in real-world settings [43]. In our case, the NGT facilitated not only the prioritization of interventions, but also their operational translation into specific products (web, social media, and types of content), reinforcing the coherence between strategic objectives and practical results.

### Interpretation of Usability Indicators and Preliminary User Perception

The results of the OCODES website usability assessment show an average SUS score above the acceptability threshold established in the literature [34], and also fall within ranges considered “excellent” according to previous interpretations of the scale [35,44]. The fact that more than 94% (94/99) of participants exceeded the SUS cutoff point of 68 reinforces the perception that the platform is easy to use, intuitive, and well-integrated from a functional point of view. However, a small proportion of respondents scored below the conventional SUS acceptability threshold, suggesting that the platform may not have been equally intuitive for all users. As no qualitative feedback, task-based usability testing, or formal accessibility assessment was conducted, the specific reasons for these lower scores cannot be determined. They may reflect individual differences in navigation preferences, expectations regarding content presentation, or other usability barriers not captured by the routine indicators used in this study.

The absence of significant differences in usability scores according to age, gender, or educational level suggests that the user experience is consistent across different sociodemographic profiles, which is a relevant finding in the field of digital health literacy. Previous studies have pointed out that digital health tools tend to reproduce usage gaps associated with age or educational level, limiting their impact on the population [45-47]. In this regard, the results suggest that the OCODES design may facilitate a relatively consistent user experience across the sociodemographic profiles represented in this initial sample.

The preliminary indicators of user perception, reflected in the high usefulness attributed to the website and in the majority’s willingness to recommend it to others, are consistent with the objective usage metrics recorded by Google Analytics. However, these findings should be interpreted as limited and operational indicators of favorable initial perception rather than as a comprehensive or validated assessment of acceptability. Although there is no “optimal” volume of visits, interactions, or growth applicable to all websites—including health websites—and the objective should focus on sustained progress aligned with the audience and the platform’s purposes [48], OCODES metrics indicate encouraging initial user engagement with the content, an important aspect of digital initiatives aimed at supporting health literacy [27,28]. However, the web analytics presented here were interpreted at an aggregate level. No stratified analysis was conducted according to content type, page category, or source platform, and therefore it is not possible to determine which specific content or channels were associated with higher or lower levels of engagement. Future evaluations should examine these metrics in a more granular way in order to identify which types of content and dissemination channels are most effective in attracting, retaining, and engaging different user groups. From a reporting perspective, these routine digital indicators should not be interpreted as the results of a formal web-based survey study. Instead, they are best understood as operational feedback and engagement indicators derived from routine platform use, whereas the NGT component of the manuscript is reported following key consensus-method reporting principles.

### Strengths and Limitations of this Study

Among the main strengths of this study are the use of a structured and transparent methodology for strategy design, as well as the diversity of profiles incorporated into NGT-2, which enriched the decision-making process and promoted attention to the needs of end users. In addition, the combination of the strategy development process with routinely generated digital indicators—including usability scores, preliminary user perception data, and website analytics—provides an initial operational view of the platform’s performance.

However, several limitations should be considered. First, the participants in the nominal groups were relatively homogeneous in terms of nationality and cultural context, which may have oriented the strategic decisions toward a Spanish-speaking population with specific sociocultural characteristics and may limit the broader transferability of the initial design. In addition, the age range represented in the nominal groups was relatively restricted, which may have reduced the incorporation of perspectives from younger or older population groups.

A further limitation concerns the composition of NGT-1. As all participants in this first nominal group were members of MEDIATICH and therefore already aligned with the program’s general mission, the panel may have been less independent than an externally constituted group. This may have influenced the framing of ideas and the prioritization of broad strategic directions during the early design stage.

Second, the indicators related to usability and user perception were derived from routine digital feedback tools and website analytics rather than from a research-specific evaluation protocol. This type of limitation has been widely described in evaluations of digital health interventions and may overestimate favorable perceptions of the initiative [12,49]. In this regard, the findings should be interpreted with caution and understood as an initial approximation of platform performance rather than as the result of a comprehensive evaluation study. In particular, preliminary user perception was explored using only 2 brief, study-specific items focused on perceived usefulness and willingness to recommend. No validated acceptability framework was applied, and no qualitative feedback, formal accessibility testing, or task-based usability evaluation was conducted. Therefore, these findings should be interpreted as preliminary operational indicators rather than as a multidimensional assessment of acceptability or user experience. Consistent with this distinction, the digital component was not treated or reported as a CHERRIES-type web survey study, whereas the NGT component was strengthened using a consensus-method reporting approach. A related but broader limitation is that the initiative was developed, implemented, and descriptively evaluated within the same academic and institutional environment, which may introduce allegiance or observer bias beyond the composition of NGT-1 alone. Several authors were involved in OCODES through their roles in SAPIENF and MEDIATICH. Although this overlap is important to acknowledge, efforts were made to improve transparency and reduce its influence, including the participation of external profiles in NGT-2, the use of predefined moderation procedures, and the incorporation of routine aggregated digital indicators alongside the narrative description of the process.

Third, the audience reached through these routine digital tools was not necessarily representative of the wider population. Users providing feedback were predominantly women and people with university education. This pattern is consistent with the literature describing greater health awareness, more active health information seeking, and greater participation in health literacy initiatives among women and individuals with higher levels of education [50,51]. Likewise, various studies have shown that these groups tend, on average, to have higher levels of health media literacy and a more critical attitude toward health information, which may explain both their greater likelihood of accessing OCODES and their more favorable perceptions of the platform [19,20]. At the same time, this profile highlights a structural challenge shared by many digital health initiatives: the difficulty of effectively reaching groups with lower educational levels, lower digital literacy, or greater social vulnerability who are often those most exposed to health misinformation [52,53]. This demographic profile may also limit the applicability of the current strategy and its preliminary indicators to more diverse populations, including culturally heterogeneous groups, men, and people with lower educational or digital literacy levels. Consequently, the present findings should be interpreted as most directly reflecting the characteristics of the audience reached in this initial phase, rather than the likely response of the broader population.

Finally, the design of this work does not allow conclusions regarding the effectiveness of OCODES in reducing health misinformation or improving health media literacy. No outcomes related to misinformation recognition, knowledge acquisition, trust in information sources, behavioral change, or exposure to false content were directly measured. Accordingly, the findings should be interpreted in terms of strategy development, routine usability indicators, preliminary user perception, and initial engagement, rather than as evidence of impact on misinformation-related outcomes.

### Practical Implications and Future Research

The results obtained support the viability of OCODES as a digital strategy for health media literacy but also point to the need to move toward more robust and diversified evaluations. Future assessments should complement routine digital feedback and web analytics with research-specific evaluation strategies, including validated acceptability frameworks, qualitative user feedback, formal accessibility assessments, and task-based usability testing to better understand how different users interact with the platform and which barriers may shape their experience. At present, this paper reports only development, routine usability indicators, preliminary user perception, and engagement, but does not assess changes in knowledge, attitudes, or behavior related to health misinformation. It is also a priority to analyze the performance of OCODES in the medium and long term, incorporating impact indicators beyond the evolution of the platform’s use, such as changes in knowledge, attitudes, or behaviors related to the identification of health misinformation, in line with the frameworks proposed for the evaluation of interventions against the infodemic [26]. Future research should incorporate robust evaluation designs to assess the impact of OCODES on misinformation-related outcomes, including (1) the ability to identify misinformation, (2) health media literacy, (3) changes in health knowledge, (4) trust in health information sources, and (5) behavioral intentions and practices. These evaluations may involve pre-post designs, controlled studies, experimental exposure to misinformation, or longitudinal follow-up approaches to determine effectiveness in real-world conditions.

At present, OCODES is primarily oriented toward Spanish-speaking users, which may limit its accessibility and relevance for linguistically and culturally diverse populations. Future development should therefore include translation and cultural adaptation strategies, as well as specific outreach actions aimed at populations with lower digital literacy, lower educational attainment, or greater social vulnerability, who are often at increased risk of exposure to health misinformation. Finally, promoting a participatory culture and combining digital and face-to-face strategies are likely to be key for expanding the reach and equity of this project as it evolves.

### Conclusions

OCODES is an innovative nurse-led initiative developed in response to an emerging public health problem through a participatory, evidence-based digital strategy. Initial results show favorable usability indicators, positive preliminary user perception, and early user engagement, supporting its feasibility as a health communication tool. However, these results do not demonstrate effectiveness in reducing health misinformation or improving health media literacy. The consolidation of OCODES as a resource with potential population-level impact will require more comprehensive evaluations, targeted strategies to reach vulnerable groups, and sustained monitoring over time.

## Supplementary material

10.2196/93910 Checklist 1 ACCORD-informed reporting checklist for the nominal group technique component.

## Abbreviations

ACCORD: Accurate Consensus Reporting Document
CHERRIES: Checklist for Reporting Results of Internet E-Surveys
NGT: nominal group technique
OCODES: Observatorio Contra la Desinformación en Salud
SUS: System Usability Scale
