# Supplementary material for: Development and Preliminary Evaluation of the OCODES Digital Strategy in Response to Health Misinformation: Nominal Group Technique and Retrospective Usability Evaluation Study
Source: JMIR Form Res. 2026 Aug 14;10:e93910. doi: 10.2196/93910 (PMC13475788; doi:10.2196/93910)
Supplement: Supplementary file 1 [file formative-v10-e93910-s001.docx]

*The table maps key ACCORD reporting domains for consensus methods to the corresponding sections of the manuscript in which the NGT design, participant recruitment, facilitation procedures, prioritisation approach, management of disagreement, and consensus thresholds are described.*

| **ACCORD reporting domain / item** | **How addressed in this manuscript** | **Manuscript section** |
| --- | --- | --- |
| Rationale for using a consensus method | The manuscript explains that NGT was selected as a structured participatory method to generate, refine, and prioritise proposals for the OCODES strategy, ensuring equitable participation and transparent decision-making. | Introduction; Methods – Conception of the OCODES Strategy |
| Type of consensus method used | Two sequential in-person Nominal Group Technique sessions were conducted (NGT-1 and NGT-2). | Methods – Conception of the OCODES Strategy |
| Aim/objective of the consensus exercise | NGT-1 aimed to identify and prioritise strategic actions for OCODES. NGT-2 aimed to translate selected priorities into concrete digital products, website sections, social media selection, content lines, and interaction mechanisms. | Abstract; Methods – First nominal group; Second nominal group |
| Participant identification and recruitment | NGT-1 participants were purposively selected MEDIATICH members with experience in health communication, healthcare practice, and health education. NGT-2 participants were purposively recruited specifically for that phase and included health professionals, communication specialists, and potential users not belonging to MEDIATICH. | Methods – First nominal group; Second nominal group |
| Participant characteristics | Demographic and profile characteristics are presented in Table 1 (NGT-1) and Table 3 (NGT-2). | Methods – Table 1; Table 3 |
| Prior involvement of participants with the initiative | The manuscript states that all NGT-1 participants were MEDIATICH members familiar with the programme’s general aims, whereas NGT-2 participants were recruited specifically for the design phase and were not members of MEDIATICH. | Methods – First nominal group; Second nominal group |
| Facilitation and moderator role | For both sessions, the moderator’s role is described as restricted to process facilitation, wording clarification, time management, and enforcement of turn-taking rules. Neutrality safeguards included avoidance of evaluative feedback and verification of reformulated items with participants before prioritisation. | Methods – First nominal group; Second nominal group |
| Generation of items/proposals | Both NGT sessions included silent idea generation followed by round-robin sharing of proposals without debate. | Methods – First nominal group; Second nominal group |
| Clarification and merging of items | The manuscript explains that overlapping ideas were reviewed collectively and merged only when participants agreed that they represented the same operational proposal. When differences in scope or meaning remained, proposals were retained as separate items. | Methods – First nominal group; Second nominal group |
| Management of disagreement | Disagreements during clarification were addressed through brief structured discussion, and unresolved distinctions were retained as separate items for prioritisation. | Methods – First nominal group; Second nominal group |
| Voting / prioritisation procedure | In both NGTs, prioritisation was conducted individually using a binary endorsement procedure (yes/no). | Methods – First nominal group; Second nominal group; Consensus criteria and analysis |
| Scoring / aggregation method | The total number of “yes” endorsements received by each proposal was counted and expressed as a proportion of the total number of participants. | Methods – Consensus criteria and analysis |
| Consensus definition | Consensus was defined a priori as support from at least 80% of participants (NGT-1: ≥12/15; NGT-2: ≥15/18). | Methods – Consensus criteria and analysis |
| Session timing and structure | The manuscript reports the duration and timing of each NGT phase (silent generation, round-robin, clarification/grouping, prioritisation, opening and closing). | Methods – First nominal group; Second nominal group |
| Reporting of prioritised outputs | The prioritised interventions and agreement values are presented in Table 4 and described narratively in the Results section. NGT-2 outputs are described as website architecture, selected social media channels, principles, thematic priorities, and interaction mechanisms. | Results – The OCODES Strategy |
| Transparency of reporting | The manuscript explicitly states that NGT reporting was aligned with key principles of ACCORD and provides this supplementary checklist to improve transparency and reproducibility. | Methods – sentence introducing Supplementary Table 1 |
